# Patterns of alcohol consumption in Brazilian adults

**DOI:** 10.1038/s41598-022-12127-2

**Published:** 2022-05-21

**Authors:** Juliana A. Plens, Juliana Y. Valente, Jair J. Mari, Gerson Ferrari, Zila M. Sanchez, Leandro F. M. Rezende

**Affiliations:** 1 Department of Psychiatry, Universidade Federal de São Paulo-UNIFESP, São Paulo, Brazil; 2 Universidad de Santiago de Chile (USACH), Escuela de Ciencias de la Actividad Física, el Deporte y la Salud, Santiago, Chile; 3 Department of Preventive Medicine, Escola Paulista de Medicina, Universidade Federal de São Paulo (UNIFESP), R. Botucatu, 740, 4° floor, São Paulo, Brazil

**Keywords:** Medical research, Epidemiology

## Abstract

In this study, we aimed to describe the patterns of alcohol consumption in Brazilian adults by sociodemographic characteristics and states according to sex. Cross-sectional study including 87,555 adults from the 2019 Brazilian National Health Survey who responded to a questionnaire on alcohol consumption and were classified as non-drinkers (0 g/day), light (1–12.5 g/day), moderate (12.6–49.9 g/day), and heavy drinkers (≥ 50 g/day). Of the Brazilian adults, 73.5% were non-drinkers. Among the drinkers, 14.8% were light drinkers. 82.6% of heavy drinkers were men. White participants drank more than non-white participants, except black women who were 38% more likely to be moderate drinkers than white women (ROR 1.38, 95% CI 1.09 to 1.76). Unmarried were more likely to be drinkers. Women over 55 and men over 65 years old were less likely to be drinkers. Compared to participants with none or incomplete primary education, both men and women with higher educational attainment were more likely to be light and moderate drinkers. The largest consumption of alcohol was found in Sergipe and Mato Grosso for men, and Mato Grosso do Sul and Bahia for women. Our findings may be useful to inform policies for reducing alcohol consumption in Brazil.

## Introduction

Alcohol consumption is one of the leading risk factors for the global burden of disease and mortality^1^. For instance, there are well-established biological mechanisms linking alcohol consumption and the risk of seven types of cancers^2,3^. In addition, alcohol is an established cause of epilepsy, poisoning, road traffic accidents, and violence^4^. Long-term chronic ethanol exposure is also associated with brain damage in humans^5^ and other psychiatric disorders such as depression, anxiety, and posttraumatic stress disorder^6,7^.

In 2016, the harmful use of alcohol was responsible for 3 million deaths worldwide (5.3% of all deaths) and 132.6 million disability-adjusted life years (DALYs) – i.e., 5.1% of all DALYs in that year^8^. In Brazil, the age-adjusted mortality rate attributable to alcohol was approximately 12.2 deaths per 100,000 inhabitants in 2016, which was higher than that in Argentina (4 per 100,000 inhabitants), similar to Chile (11.6 per 100,000 inhabitants) and lower than that in Mexico (17 per 100,000 inhabitants)^9^. In the same year, Brazilians over 15 years old had an alcohol per capita consumption (APC) of 7.5 L of pure alcohol per year, but the APC among drinkers (alcohol consumption divided by people over 15 years old who referred to drinking) was 19.2 L of pure alcohol per year. The APC among drinkers was, approximately, 150% higher than that of the general population over 15 years of age in Brazil**.** Drinkers in Brazil consumed atypically large volumes of alcohol as compared to those in other countries in the Americas, such as USA (13.2 L of pure alcohol per year) and Argentina (14.6 L of pure alcohol per year)^10^.

Sociodemographic characteristics such as sex, age, and skin color are associated with different patterns of alcohol consumption, and these associations may differ between countries^11–14^. Men are more likely to drink and have more alcohol-related health and social issues than women^10,15,16^. Compared to men, women are more likely to be lifetime abstainers or drink less, and less likely to experience drinking problems and alcohol-related disorders^15–17^. However, biological factors in alcohol pharmacokinetics make women more likely to have medical problems^18^. Heavy drinking may be motivated by cultural factors related to the social roles of men and women in society^19^. Younger adults are also more likely to be involved in dangerous patterns of alcohol consumption and other harmful behaviors than older adults in wealthier countries^20^.

The most up-to-date epidemiological data on drinking behavior in the Brazilian population were collected in 2019^21^, just before the beginning of the Covid-19 pandemic. The National Health Survey 2019 (*Pesquisa Nacional de Saúde*—PNS) data was used in a recent study aimed at characterizing heavy drinking behavior in the Brazilian population^21^. They found a higher prevalence of heavy drinking in young adults, men, single, black, and less educated^21^. In addition, a significant increase in heavy drinking behavior was noticed between 2013 and 2019, particularly in the younger age group, white and brown, single, and women aged 30–44 years^21^. People with complete elementary school or incomplete high school education, and those who reported having completed high school, incomplete higher education or higher education also showed a significant increase in heavy drinking behavior in the same period^21^. Maintenance of a reduction trend in the percentage of heavy drinkers with increasing age was noticed when comparing the two editions of PNS^21^.

Understanding not only heavy drinking, but also other drinking patterns (non-drinkers, light, moderate, and heavy drinkers) by sociodemographic factors in the Brazilian population may be helpful to inform policies and interventions to reduce alcohol consumption. This might be as there is no minimum amount of alcohol considered safe for human consumption^22,23^. For instance, different categories of alcohol consumption, from light to heavy drinking, are associated with an increased risk of cancer in a dose-dependent manner^24^. In addition, the World Health Organization (WHO) suggests regular publication of national reports on alcohol consumption^25^ as there have been several fluctuations in the data on alcohol consumption over the years in many countries. Of note, information on all patterns of alcohol use, not only those on the extreme, is needed to adequately address public policies. Another important point is the lack of information regarding the geographic distribution of alcohol consumption, as Brazil is one of the largest countries in the world, with important cultural differences as well as economic and health inequalities.

In this study, we aimed to describe the patterns of alcohol consumption (non-drinkers, light, moderate, and heavy drinkers) in Brazilian adults in 2019, using data from 87,555 participants from the PNS 2019, the largest and most recent nationally representative household survey conducted in the country. We also examined the associations of sociodemographic characteristics and states with patterns of alcohol consumption in Brazilian adults according to sex.

## Methods

All methods were performed in accordance with STROBE guideline (Table S3)^26^.

### Study design and sample

This study used cross-sectional data from the PNS 2019, a national household survey conducted by the Brazilian Ministry of Health in partnership with the Brazilian Institute of Geography and Statistics (Instituto Brasileiro de Geografia e Estatística—IBGE). PNS 2019 examined health status perception, lifestyle, and noncommunicable diseases, in addition to providing demographic and socioeconomic information. The PNS 2019 sample originated from the Master Sample of IBGE's Integrated Home Research System (SIPD), which was selected in three stages. In the first stage, 8,036 census tracts (primary sampling units) were randomly selected based on the IBGE census (53% of PSU within the quantity of 15,096 PSU, which corresponds to a quarter of the Master Sample), with a probability proportional to the size of the subsample. In the second stage, households were selected from a national registry of addresses using simple random sampling. Finally, in the third stage, persons aged 15 years or older were selected from households by simple random sampling. Further methodological details on PNS 2019 were described elsewhere^27^*.*

The selected sample consisted of 108,525 households and 94,114 participants who were interviewed^28^, with an overall response rate of 93.6%. All participants were informed on the study’s purpose and agreed to participate by signing a free and informed written consent form^29^. In this study, we included adults aged ≥ 18 years with complete information on alcohol consumption (N = 87,555).

### Ethics

The study was performed in accordance with Declaration of Helsinki. PNS 2019 was approved by the Brazilian National Commission on Ethics in Research (CONEP) of the National Health Council (CNS) under Process 3,529,376, issued on August 23, 2019^27^. CONEP is the maximum instance of ethical evaluation in research protocols involving human beings in Brazil and has autonomy for the ethical analysis of highly complex research protocols, and in research projects proposed by the Ministry of Health. All participants were consulted, educated on the study’s purpose and agreed to participate by signing a free and informed written consent form^29^.

### Instrument and measures

Trained interviewers collected information on sociodemographic characteristics and lifestyle risk factors through handheld computers (personal digital assistance) in face-to-face interviews.

#### Patterns of alcohol consumption

Alcohol consumption was measured through a questionnaire about the weekly frequency of alcoholic beverage use and the number of drinks consumed on each day they reported drinking. The questions about alcohol included in the questionnaire were formulated as follows: “How often do you consume alcoholic beverages?” and “How many days a week do you usually consume alcoholic beverages?” and the amount of alcohol used was collected through: “In general, on the day you drink, how many drink units do you ingest?”. Participants were instructed about the equivalence of the drink unit as follows: one drink unit is equivalent to one can of beer, one glass of wine, or one dose of cachaça, whiskey, or any other distilled drink. One bottle of beer was converted into two cans of beer or two drink units^30^. We used the number of days the person mentioned drinking in a week multiplied by the number of drink units ingested by the person. The results were divided by seven to generate the average consumption of alcohol in drink units per day.

The average consumption of alcohol in drink units per day was then multiplied by grams of pure ethanol as a standard measure of ethanol intake, using the equivalence 12.5 g of alcohol in a drink unit^24^. The patterns of alcohol consumption (in grams per day: g/day) of pure alcohol consumed in the past 30 days were categorized into four categories: non-drinkers (0 g/day), light drinkers (1–12.5 g/day), moderate drinkers (12.6–49.9 g/day), and heavy drinkers (≥ 50 g/day)^24^. Those who reported never drinking or drinking less than once a month were considered non-drinkers.

#### Sociodemographic characteristics

Information on sex (men and women), skin color (white, black, brown, yellow, indigenous), age group (18–24, 25–34, 35–44, 45–54, 55–64, ≥ 65 years), marital status (married or not married) and educational attainment (none or incomplete primary education, complete primary or incomplete secondary education, complete secondary or incomplete undergraduate course and university graduate) and the federal units of residence (26 states and the Federal District) were included in the sociodemographic questionnaire.

#### Statistical analyses

Sociodemographic characteristics were described as percentages and 95% confidence intervals (95% CI) according to patterns of alcohol consumption. Multinomial multivariable logistic regression models were performed to estimate the relative odds ratios (RORs) and its 95% CIs for the association between sociodemographic characteristics (age group, skin color, marital status, and educational attainment) and patterns of alcohol consumption by sex.

All statistical analyses were performed using Stata 14 with the svyset command to account for the complex sampling design. Microsoft Excel Visual Basic for Applications was used to create the choropleth maps of the average consumption of alcohol in grams per day according to Brazilian state and the Federal District by sex. The dataset and questionnaire are available on the PNS website of the Oswaldo Cruz Foundation (www.pns.fiocruz.br).

## Results

### Characteristics of the participants according to patterns of alcohol consumption

Women comprised 53.2% of the Brazilian population. Approximately 43% were white and 43% brown, 56% were not married, and 20.2% were aged between 35 and 44 years old. Approximately 35% had no or incomplete primary education, and 35% had complete secondary education or incomplete undergraduate degrees. Non-drinkers accounted for 73.5% of the population. Among the drinkers, most were men, not married, white and brown and had complete secondary or incomplete undergraduate education, except heavy drinkers that were brown and had none or incomplete primary education. Regarding the age group, most of the drinkers were aged between 25 and 44 years (Table 1).

**Table 1:** Characteristics of the participants according to patterns of alcohol consumption (N = 87,555).

Variables	Total		Non-drinkers^a^		Light drinkers^b^		Moderate drinkers^c^		Heavy drinkers^d^	
	N = 87,555 100%		N = 64,306 73.5%		N = 12,959 14.8%		N = 8,699 9.9%		N = 1,591 1.8%	
	%	95% CI	%	95% CI	%	95% CI	%	95% CI	%	95% CI
**Sex**										
Men	46.8	46.2–47.4	40.0	39.6–40.4	57.4	55.9–58.9	75.2	73.7–76.7	82.6	79.3–85.5
Women	53.2	52.6–53.8	60.0	59.6–60.4	42.6	41.1–44.2	24.8	23.3–26.3	17.4	14.5–20.7
**Skin color**										
White	43.3	42.6–44.0	41.6	40.8–42.3	53.1	51.3–54.8	42.6	40.7–44.5	36.3	31.5–41.5
Black	11.5	11.1–11.9	11.4	10.9–11.8	10.6	9.5–11.8	12.9	11.8–14.1	13.8	11.4–16.7
Yellow	0.9	0.8–1.1	1.0	0.9–1.2	0.7	0.5–1.0	0.8	0.4–1.2	0.5	0.2–1.2
Brown	43.8	43.1–44.5	45.5	44.8–46.2	35.3	33.7–36.9	43.2	41.3–45.1	48.8	43.9–53.7
Indigenous	0.5	0.4–0.6	0.5	0.5–0.6	0.3	0.2–0.5	0.5	0.3–0.9	0.6	0.3–1.2
**Marital status**										
Married	44.0	43.4–44.6	45.6	45.0–46.3	43.5	42.0–45.1	36.3	34.2–38.4	22.8	19.6–26.5
Not married	56.0	55.4–56.6	54.4	53.7–55.0	56.5	54.9–58.0	63.7	61.7–65.8	77.2	73.5–80.4
**Educational attainment**										
None or incomplete primary education	34.8	34.2–35.4	38.3	37.7–39.1	22.8	21.5–24.1	26.4	24.7–28.2	34.9	30.5–39.6
Complete primary or incomplete secondary education	14.1	13.7–14.5	13.7	13.2–14.2	13.2	12.1–14.5	17.2	15.7–18.7	21.7	18.0–25.9
Complete secondary or incomplete undergraduate course	35.2	34.6–35.7	34.1	33.4–34.8	37.7	36.1–39.4	39.4	37.5–41.3	34.3	29.8–39.0
University graduate	15.9	15.3–16.6	13.9	13.3–14.5	26.3	24.5–28.1	17.0	15.6–18.5	9.1	6.9–12.0
**Age (in years)**										
18–24	12.3	11.8–12.8	12.9	12.6–13.3	15.7	14.5–17.1	16.3	14.8–18.0	23.3	18.7–28.7
25–34	18.4	17.9–18.9	16.7	16.3–17.1	20.4	19.1–21.7	23.7	22.2–25.3	24.7	21.2–28.5
35–44	20.6	20.1–21.1	19.5	19.0–20.0	21.1	19.9–22.4	23.8	22.2–25.6	24.0	20.6–27.8
45–54	18.2	17.7–18.7	17.9	17.5–18.3	17.7	16.6–18.9	18.4	16.5–20.4	14.9	12.0–18.3
55–64	15.3	14.9–15.7	15.6	15.2–16.0	15.0	13.8–16.1	12.1	11.0–13.3	9.5	7.6–11.8
≥ 65	15.2	14.7–15.6	17.4	17.1–17.7	10.1	9.3–10.9	5.7	4.9–6.4	3.6	2.5–5.3

Results include sample weights and control for survey design.

^a^Defined as reporting having 0 g/day of pure alcohol in the past 30 days.

^b^Defined as reporting having between 1 and 12.5 g per day of pure alcohol in the past 30 days.

^c^Defined as reporting having between 12.6 and 49.9 g per day of pure alcohol in the past 30 days.

^d^Defined as reporting having between ≥ 50 g per day of pure alcohol in the past 30 days.

### Patterns of alcohol consumption by sociodemographic characteristics in Brazilian adults

**Figure 1:**
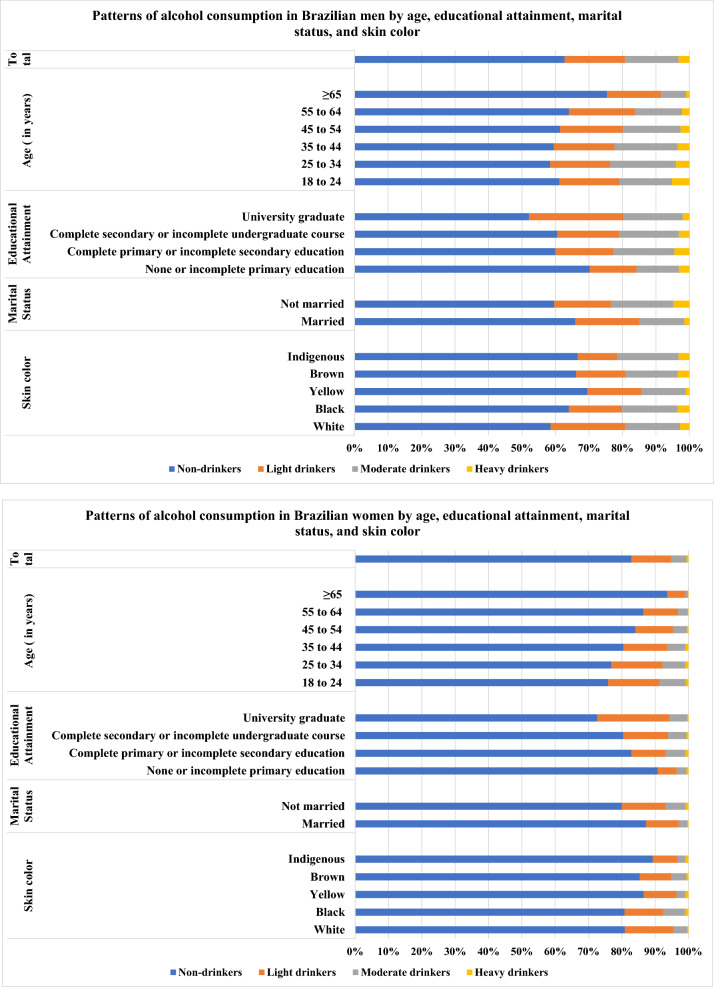
Patterns of alcohol consumption by sociodemographic characteristics in Brazilian adults. These figures show the patterns of alcohol consumption by sociodemographic characteristics according to sex. The prevalence corresponding to each pattern of alcohol consumption (non-drinker, light, moderate and heavy drinker) are presented in horizontal bars. The first line of each figure shows prevalence of patterns of alcohol consumption for the total sample of men or women, and the subsequent lines show prevalence for each sociodemographic characteristic: age, educational attainment, marital status and skin color.

Figure 1 shows the patterns of alcohol consumption according to sex. The majority of men (62.7%) and women (82.9%) were non-drinkers. In men, the prevalence of light drinkers was 18.1%, followed by 16.0% of moderate drinkers, and 3.2% of heavy drinkers. In women, the prevalences were 11.9% for light drinkers, 4.6% for moderate drinkers and 0.6% for heavy drinkers. Among adults who reported drinking, the majority were light drinkers, except men aged between 25 and 44 years old, non-white, with complete primary or incomplete secondary education, and not married, who were more likely to be moderate drinkers. Detailed data referred to in Fig. 1 can be found in Supplementary Tables S1 and S2.

### Average alcohol consumption (grams per day) in Brazilian adults in 2019 by state according to sex

**Figure 2:**
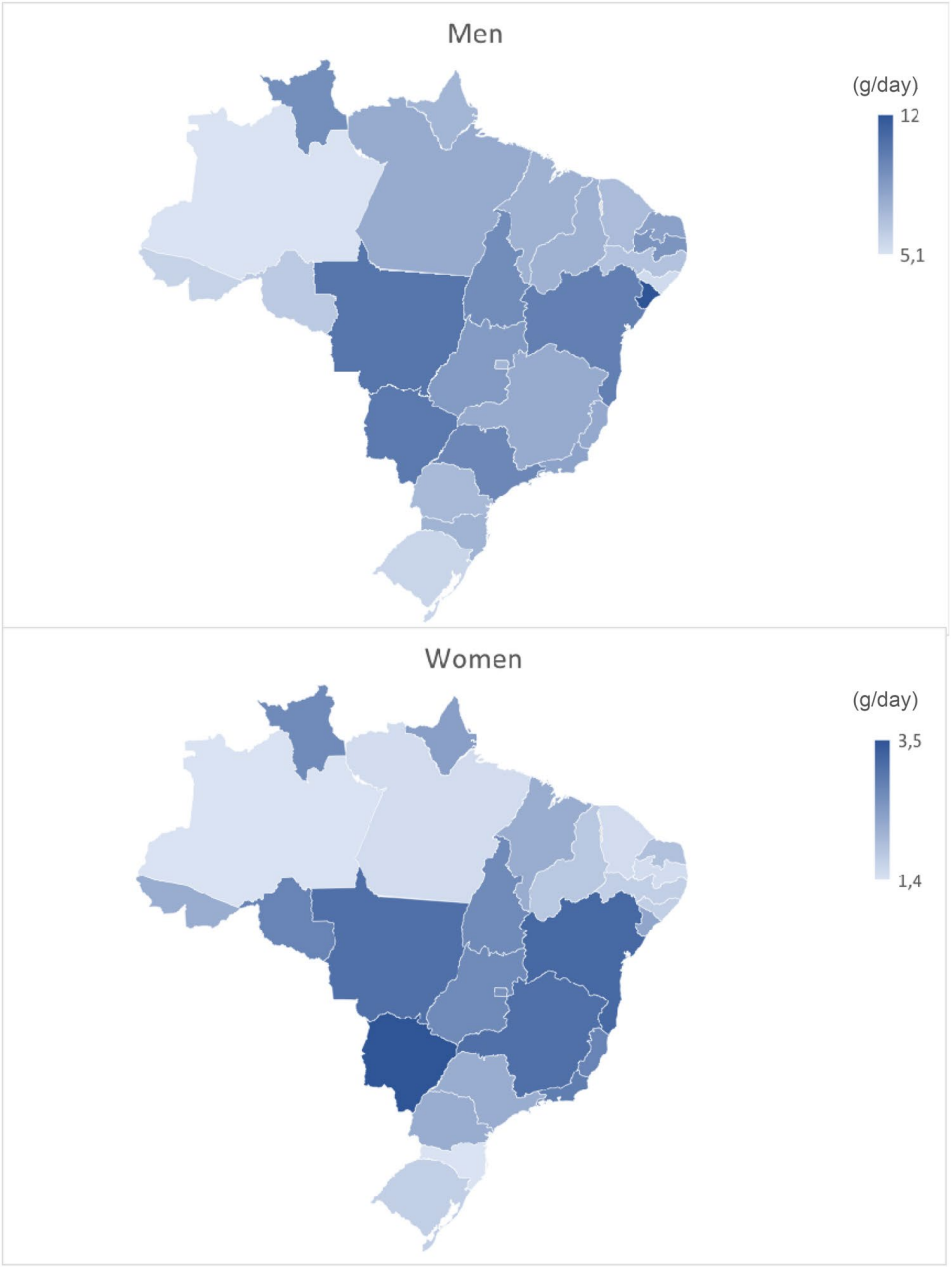
Average alcohol consumption (grams per day) by Brazilian adults in 2019 by state, by sex. These figures present the average consumption of alcohol in grams per day (g/day) by Brazilian states and Federal District, by sex. The maps of Brazil show the states in shades of blue according to the average consumption of alcohol for men and women. To the right of the maps are the blue scales corresponding to consumption: the darker, the higher the average consumed.

Figure 2 presents the average consumption of alcohol in grams per day (g/day) by individuals in Brazilian states and the Federal District, according to sex. The highest average amounts of alcohol consumed among men were in Sergipe (12.0 g/day) and Mato Grosso (10.4 g/day). In women, the highest amounts consumed were in Mato Grosso do Sul (3.5 g/day) and Bahia (3.2 g/day).

### Association between sociodemographic characteristics and patterns of alcohol consumption by sex

Multinomial multivariable logistic regression models for the association between sociodemographic characteristics and patterns of alcohol consumption by sex are presented in Table 2. The ROR were obtained using non-drinkers as the reference category. Non-white Brazilians drank less than white Brazilians, except black women who were 38% more likely to be moderate drinkers than white women (ROR 1.38, 95% CI 1.09–1.76). Compared to married counterparts, unmarried men were more likely to be moderate and heavy drinkers (ROR 1.54, 95% CI 1.39–1.71 and ROR 2.88, 95% CI 2.29–3.63, respectively) and unmarried women were more likely to drink at all (light: ROR 1.62, 95% CI 1.45–1.81, moderate: ROR 2.43, 95% CI 2.01–2.92, and heavy drinkers: ROR 2.54, 95% CI 1.65–3.89). Compared to adults aged between 18 and 24 years, women older than 55 years as well as men over 65 years of age were less likely to be drinkers. On the other hand, younger men aged between 25 and 54 years were more likely to be moderate drinkers (25–34 y.o: ROR 1.46, 95% CI 1.20–1.78; 35–44 y.o: ROR 1.56, 95% CI 1.27–1.91; and 45–54 y.o: ROR 1.49, 95% CI 1.20–1.86). Compared to people with none or incomplete primary education, both men and women with higher educational attainment were more likely to be light and moderate drinkers and women who graduated from college were less likely to be heavy drinkers (ROR 0.43, 95% CI 0.21–0.87) (Table 2).

**Table 2:** Multivariable multinomial logistic regression for the association between sociodemographic characteristics and patterns of alcohol consumption by sex (N = 87,555).

Variables	Men n = 41,184									Women n = 46,371								
	Light drinkers^b^ × non-drinkers^a^			Moderate drinkers^c^ × non-drinkers^a^			Heavy drinkers^d^ × non-drinkers^a^			Light drinkers^b^ × non-drinkers^a^			Moderate drinkers^c^ × non-drinkers^a^			Heavy drinkers^d^ × non-drinkers^a^		
	ROR	95% CI	*p* value	ROR	95% CI	*p* value	ROR	95% CI	*p* value	ROR	95% CI	*p* value	ROR	95% CI	*p* value	ROR	95% CI	*p* value
**Skin color**																		
White	Ref			Ref			Ref			Ref			Ref			Ref		
Black	0.71	0.59–0.85	< 0.001	0.90	0.77–1.05	0.170	0.93	0.68–1.28	0.665	0.87	0.71–1.06	0.171	1.38	1.09–1.76	0.009	1.77	0.98–3.21	0.059
Yellow	0.45	0.29–0.71	0.001	0.65	0.33–1.26	0.200	0.33	0.13–0.82	0.018	0.58	0.31–1.07	0.079	0.55	0.27–1.11	0.096	2.15	0.46–9.99	0.331
Brown	0.64	0.58–0.71	< 0.001	0.83	0.74–0.93	0.001	0.91	0.70–1.18	0.471	0.66	0.59–0.74	< 0.001	0.94	0.79–1.11	0.462	1.10	0.65–1.86	0.721
Indigenous	0.51	0.29–0.90	0.019	0.94	0.56–1.58	0.812	0.86	0.38–1.94	0.714	0.54	0.21–1.35	0.186	0.49	0.24–0.99	0.048	1.64	0.35–7.74	0.532
**Marital status**																		
Married	Ref			Ref			Ref			Ref			Ref			Ref		
Not married	1.05	0.95–1.17	0.336	1.54	1.39–1.71	< 0.001	2.88	2.29–3.63	< 0.001	1.62	1.45–1.81	< 0.001	2.43	2.01–2.92	< 0.001	2.54	1.65–3.89	< 0.001
**Educational attainment**																		
None or incomplete primary education	Ref			Ref			Ref			Ref			Ref			Ref		
Complete primary or incomplete secondary education	1.38	1.18–1.61	< 0.001	1.47	1.25–1.73	< 0.001	1.21	0.88–1.66	0.242	1.63	1.34–1.97	< 0.001	1.49	1.17–1.91	0.001	1.12	0.64–1.96	0.699
Complete secondary or incomplete undergraduate course	1.42	1.26–1.61	< 0.001	1.40	1.22–1.60	< 0.001	0.81	0.61–1.09	0.166	2.05	1.76–2.38	< 0.001	1.31	1.06–1.61	0.011	0.62	0.36–1.05	0.074
University graduate	2.39	2.07–2.76	< 0.001	1.64	1.40–1.91	< 0.001	0.79	0.54–1.17	0.246	3.76	3.19–4.42	< 0.001	1.81	1.44–2.27	< 0.001	0.43	0.21–0.87	0.020
**Age (years)**																		
18–24	Ref			Ref			Ref			Ref			Ref			Ref		
25–34	0.97	0.79–1.18	0.747	1.46	1.20–1.78	< 0.001	1.02	0.69–1.50	0.925	0.99	0.81–1.22	0.941	1.00	0.78–1.29	0.992	1.44	0.88–2.35	0.149
35–44	1.01	0.82–1.24	0.935	1.56	1.27–1.91	< 0.001	1.03	0.70–1.51	0.882	0.88	0.73–1.07	0.214	0.86	0.67–1.10	0.231	1.36	0.77–2.40	0.295
45–54	1.06	0.86–1.31	0.584	1.49	1.20–1.86	< 0.001	0.81	0.52–1.27	0.357	0.83	0.67–1.02	0.070	0.68	0.51–0.91	0.010	0.66	0.35–1.24	0.197
55–64	1.10	0.87–1.39	0.410	1.21	0.98–1.50	0.081	0.67	0.43–1.05	0.081	0.77	0.62–0.95	0.016	0.45	0.34–0.61	< 0.001	0.24	0.12–0.50	< 0.001
≥ 65	0.79	0.63–0.99	0.045	0.58	0.46–0.74	< 0.001	0.25	0.14–0.45	< 0.001	0.41	0.32–0.52	< 0.001	0.13	0.09–0.20	< 0.001	0.04	0.01–0.12	< 0.001

Results include sample weights and control for survey design.

^a^Defined as reporting having 0 g/day of pure alcohol in the past 30 days.

^b^Defined as reporting having between 1 and 12.5 g per day of pure alcohol in the past 30 days.

^c^Defined as reporting having between 12.6 and 49.9 g per day of pure alcohol in the past 30 days.

^d^Defined as reporting having between ≥ 50 g per day of pure alcohol in the past 30 days.

## Discussion

In this study, we provided a comprehensive description of the patterns of alcohol consumption in 87,555 Brazilian adults according to sociodemographic characteristics and regional distribution across the country. The prevalence of non-drinkers was 73.5%. The highest average of alcohol consumption was found in Sergipe and Mato Grosso among men, and Mato Grosso do Sul and Bahia among women. Drinkers were mostly men, especially heavy drinkers. Women over 55 years of age and men over 65 years of age were less likely to be drinkers and men between 18 and 54 years were more likely moderate drinkers. Compared to people with none or incomplete primary education, both men and women with higher educational attainment were more likely to be light and moderate drinkers. White participants drank more than non-white participants, except black women.

We found a higher prevalence of heavy drinkers in men than women. Our study showed a higher prevalence of heavy drinkers in men (3.2%) than in women (0.6%), which is in line with previous studies conducted in Brazil and other countries in the Americas^10,21,31,32^. Of note, we found that Brazilians consume larger volumes of alcohol than citizens of other countries in the Americas^8^. A study showed that differences in the habit of heavy drinking can be related with the social role of the men and the women in the society, what could justify higher consumption by men^19^. A possible explanation for this is that the symbol of masculinity expose male sex more to alcohol use^33^. These findings highlight the importance of public policies that target the reduction of alcohol consumption among men. Especially because men that are more likely than women to take other risks behaviors, that when combined with alcohol, can lead to increased risk of health problems^34^.

Alcohol is an established cause of cancer, as well as communicable (e.g., tuberculosis and HIV/AIDS) and other noncommunicable diseases (e.g., liver cirrhosis, breast cancer, and cardiovascular disease), violence, injury, and neuropsychiatric disorders, hence the need for the implementation of population-wide strategies to reduce alcohol consumption, such as national alcohol excise taxes, regulations of hours of sale, and advertising restrictions, as suggested by the WHO^35^.

We also found a significant variation in the patterns of alcohol consumption across Brazilian states and no trend towards higher consumption in states with weaker policies and lower consumption in states with stronger policies. The highest average alcohol consumption in men was found in Sergipe and Mato Grosso, while women from Mato Grosso do Sul and Bahia had the highest average consumption of alcohol. A recent study showed differences in alcohol policies across states in Brazil, and despite the autonomy to complement federal laws, the states have not yet addressed the most important gaps in alcohol regulation policies^36^. The main gaps include pricing policies, physical availability of alcohol, and comprehensive marketing regulation^36^. Some states such as Mato Grosso do Sul have not effectively complemented these policies, even though they can legally do so^36^ which is in agreement with our study that shows that Mato Grosso do Sul has one of the highest average amounts of alcohol consumed among both men and women. Amazonas, in turn, had medium levels of alcohol policies (as the rest of Brazil) according to 2021 data from Oliveira et al.^36^ and showed lower levels of consumption in our findings. This variation may reflect not only differences in enforcement of existing policies controlling alcohol use, but also variations in state sociodemographics such as age composition, skin color, educational attainment, and economic factors^16^. For several reasons, it is not easy to directly correlate state alcohol policies with alcohol consumption in Brazil.

There is no evidence that these policies could explain the differences in alcohol consumption among men and women in the states. ﻿States such Minas Gerais that have enacted federal laws to complement and close the federal gap in this domain, establishing objective guidelines for the care of women with alcohol and drug problems, did not present a low average of alcohol consumption in women. Although the prevalence of alcohol consumption in women has decreased in most parts of the world, in absolute terms, there is an increase in the number of women consuming alcoholic beverages^8^. Thus, variation among the sexes may be explained by other factors such as biological and social differences and variation in state populations by age, educational attainment, economic factors, and cultural factors. Data show that the accelerated or compressed progression of alcohol-related problems and their consequences observed in women relative to men highlight sex differences in the pharmacokinetics, pharmacodynamics, cognitive, and psychological consequences of alcohol^18^. In addition, differences in alcohol effects on behavior may also be driven by psycho-socio-cultural (sex-related) factors^19^. Findings from our large, representative survey with a country-wide territorial coverage provide important descriptive information on patterns of alcohol consumption by geographic area, which may be useful to identify areas and populations where alcohol control efforts should be targeted.

Multivariable models indicated that alcohol consumption was also differently distributed between skin colors in Brazil. Overall, non-white people usually drink less than whites, except black women who are 38% more likely to be moderate drinkers (people who drink 12.6–49.9 g/day of pure alcohol). These data could be due to the direct and indirect effects of skin color on income, even when we hold other variables constant, including social origin^37^. People with higher socioeconomic status may consume similar or greater amounts of alcohol compared to people with lower socioeconomic status, although the lower socioeconomic status experiences a worse burden of negative alcohol-related consequences^38^. Compared to whites who share similar characteristics, such as geographic region, age, sex, and socioeconomic status, blacks tend to experience further disadvantages caused by racism. Racism has a direct effect on reduced income of blacks by 7.4%, but also indirect effects through lower education and occupation. In total, blacks tend to have an income that is 16.8% lower than whites^37^. Our findings that black women are 38% more likely to engage in moderate episodic drinking than white women align with this evidence. This race/ethnic inequality in alcohol use has also been shown in previous studies conducted in Brazil and other countries^31,39^. A study conducted using data from the National Alcohol Survey in the United States indicated that black women had a higher risk of dependence at all levels of heavy drinking^39^, highlighting the sex-specific nature of racial/ethnic disparities (intersectionality).

Our study also showed that not being married was positively associated with greater alcohol consumption, regardless of sex and other sociodemographic characteristics. These findings are consistent with studies showing that married adults are less likely to drink than their single or divorced counterparts^40^. Being never married, divorced, or separated is a strong predictor of hazardous alcohol consumption behaviors^41^. Consumption of alcohol is higher among unmarried adults, which could be explained by the fact that alcohol is a licit drug related to sociability^42^.

We found that women aged > 55 years and men aged > 65 years were less likely to be drinkers. On the other hand, younger men (aged between 25 and 54 years) were more likely to be moderate drinkers. In PNS 2013, the high prevalence rates of heavy drinking stood out among younger individuals (18–29; 30–44 years old), however they did not find a difference between men and women^32^. As alcohol is a leading cause of premature death, lower alcohol consumption in older participants may be related to survivorship bias^43^. Another possible explanation to findings related to this difference between age groups suggests that alcohol expectancies decrease with increasing age. Younger adults share positive alcohol expectancies related to social assertiveness and sexual enhancement differently from older adults^44^. In this context, the findings indicate the importance to implement evidence-based strategies to reduce alcohol use among Brazilian young adults, with special emphasis in young men^45^.

We also found that both men and women with higher educational attainment were more likely to be light and moderate drinkers, when compared to people with none or incomplete primary education. This finding is in line with other Brazilian studies showing that high socioeconomic status seems to be associated with higher risk to engage in alcohol consumption, which may be explained by the greater purchasing power in this population^32^. Some data highlight that educational attainment is shaped by social class inequalities^46^ and some studies have shown that alcohol abuse has increased in some state capitals with a greater representation of higher socioeconomic status^47^. Approximately 65% of the social origin effect on income occurs indirectly, especially through education^37^. Brazilians of higher socioeconomic status and education should have a higher average consumption of alcohol than those with lower socioeconomic status. However, our study showed that women who graduated from college were less likely to be heavy drinkers and no significant association was found between educational attainment and heavy drinking among men. PNS 2013 also found that educational attainment was a factor negatively related to the excessive episodic use of alcohol among women^32^. This finding may reflect the complex relationship between education, income and drinking patterns. Evidence suggests that people with higher educational attainment tends to have a lower prevalence of alcohol dependence^48^. This might be explained by studies that indicate that completing more years of education is linked to health benefits after leaving school, such as better health insurance, access to medical care, timely health checkups and the resources to live a healthier lifestyle and to reside in healthier homes and neighborhoods^49–51^. Further investigation is needed in order to better understand the association between alcohol use and educational attainment.

There are some limitations to this study that should be considered. Alcohol consumption was self-reported in a face-to-face interview, which may have underestimated the actual amount of alcohol consumed. Consequently, the misclassification of participants across categories of alcohol consumption may have occurred.

In conclusion, our study identified sociodemographic characteristics associated with different patterns of alcohol consumption in Brazil. We found that, approximately, 26.5% of the Brazilian population were drinkers, among whom 1.8% were heavy drinkers. Most of the heavy drinkers were men. Non-white people drink less than whites, except black women who were 38% more likely to be moderate drinkers than white women. Women over 55 and men over 65 years old were less likely to be drinkers. Compared to people with none or incomplete primary education, both men and women with higher educational attainment were more likely to be light and moderate drinkers. Finally, the highest average consumption of alcohol in men was found in Sergipe and Mato Grosso, while in women was in Mato Grosso do Sul and Bahia. These findings may be useful to inform policies and strategic interventions to reduce alcohol consumption in Brazil.

## Supplementary Information

Supplementary Tables.
