# Supplementary material for: Patterns of alcohol consumption in Brazilian adults
Source: Sci Rep. 2022 May 21;12:8603. doi: 10.1038/s41598-022-12127-2 (PMC9123624; doi:10.1038/s41598-022-12127-2)
Supplement: Supplementary file 1 — Supplementary Tables. [file 41598_2022_12127_MOESM1_ESM.doc]

**Supplementary Table S1: Patterns of alcohol consumption in Brazilian men by age, educational attainment, marital status and skin color**

| Variables |  | Non-drinkers^1^ | | Light drinkers^2^ | | Moderate drinkers^3^ | | Heavy drinkers^4^ | |
| --- | --- | --- | --- | --- | --- | --- | --- | --- | --- |
|  |  | % | 95%CI | % | 95%CI | % | 95%CI | % | 95%CI |
|  | Total | 62.7 | 61.9 to 63.6 | 18.1 | 17.4 to 18.8 | 16.0 | 15.4 to 16.7 | 3.2 | 2.8 to 3.5 |
|  |  |  |  |  |  |  |  |  |  |
| Age (in years) |  |  |  |  |  |  |  |  |  |
|  | 18 to 24 | 61.2 | 58.2 to 64.1 | 18.0 | 15.9 to 20.4 | 15.6 | 13.7 to 17.8 | 5.2 | 3.8 to 7.0 |
|  | 25 to 34 | 58.4 | 56.3 to 60.4 | 17.9 | 16.4 to 19.6 | 19.7 | 18.2 to 21.3 | 4.0 | 3.3 to 4.8 |
|  | 35 to 44 | 59.5 | 57.6 to 61.4 | 18.1 | 16.7 to 19.5 | 18.9 | 17.5 to 20.4 | 3.5 | 2.9 to 4.2 |
|  | 45 to 54 | 61.4 | 59.2 to 63.5 | 18.7 | 17.0 to 20.4 | 17.3 | 15.2 to 19.7 | 2.6 | 2.1 to 3.4 |
|  | 55 to 64 | 64.0 | 62.0 to 66.1 | 19.7 | 17.8 to 21.6 | 14.1 | 12.7 to 15.6 | 2.2 | 1.7 to 2.8 |
|  | ≥ 65 | 75.4 | 73.5 to 77.2 | 16.1 | 14.7 to 17.7 | 7.5 | 6.5 to 8.6 | 1.0 | 0.6 to 1.5 |
| Educational attainment |  |  |  |  |  |  |  |  |  |
|  | None or incomplete primary education | 70.2 | 68.9 to 71.5 | 14.0 | 13.1 to 14.9 | 12.7 | 11.7 to 13.8 | 3.1 | 2.6 to 3.7 |
|  | Complete primary or incomplete secondary education | 60.0 | 57.7 to 62.2 | 17.3 | 15.5 to 19.3 | 18.2 | 16.5 to 20.2 | 4.5 | 3.6 to 5.6 |
|  | Complete secondary or incomplete undergraduate course | 60.6 | 59.0 to 62.2 | 18.5 | 17.3 to 19.8 | 17.8 | 16.6 to 19.0 | 3.1 | 2.5 to 3.7 |
|  | University graduate | 52.1 | 49.8 to 54.4 | 28.3 | 26.2 to 30.5 | 17.6 | 16.1 to 19.2 | 2.0 | 1.5 to 2.8 |
| Marital status |  |  |  |  |  |  |  |  |  |
|  | Married | 65.9 | 64.8 to 67.1 | 19.2 | 18.1 to 20.3 | 13.4 | 12.5 to 14.3 | 1.5 | 1.3 to 1.8 |
|  | Not married | 59.6 | 58.4 to 60.9 | 17.1 | 16.2 to 18.1 | 18.5 | 17.6 to 19.5 | 4.8 | 4.2 to 5.4 |
| Skin Color |  |  |  |  |  |  |  |  |  |
|  | White | 58.5 | 57.1 to 59.9 | 22.4 | 21.2 to 23.7 | 16.3 | 15.3 to 17.4 | 2.8 | 2.3 to 3.4 |
|  | Black | 64.0 | 61.7 to 66.4 | 15.9 | 14.0 to 18.1 | 16.6 | 14.9 to 18.4 | 3.5 | 2.8 to 4.4 |
|  | Yellow | 71.6 | 62.2 to 79.4 | 13.6 | 9.4 to 19.5 | 13.7 | 7.7 to 23.3 | 1.1 | 0.4 to 2.5 |
|  | Brown | 66.2 | 65 to 67.4 | 14.7 | 13.8 to 15.7 | 15.6 | 14.7 to 16.6 | 3.5 | 3.0 to 4.0 |
|  | Indigenous | 66.6 | 57.6 to 74.5 | 11.9 | 7.2 to 19.2 | 18.3 | 11.6 to 27.7 | 3.2 | 1.5 to 6.6 |

Results include sample weights and control for survey design.

1 Defined as reporting having 0 g/day of pure alcohol in the past 30 days.

2 Defined as reporting having between 1 to 12.5 grams per day of pure alcohol in the past 30 days.

3 Defined as reporting having between 12.6 to 49.9 grams per day of pure alcohol in the past 30 days.

4 Defined as reporting having between ≥50 grams per day of pure alcohol in the past 30 days.

**Supplementary Table S2: Patterns of alcohol consumption in Brazilian women by age, educational attainment, marital status and skin color**

| Variables |  | Non-drinkers^1^ | | Light drinkers^2^ | | Moderate drinkers^3^ | | Heavy drinkers^4^ | |
| --- | --- | --- | --- | --- | --- | --- | --- | --- | --- |
|  |  | |  | |  | |  | |
|  |  | % | 95%CI | % | 95%CI | % | 95%CI | % | 95%CI |
|  | Total | 82.9 | 82.2 to 83.6 | 11.9 | 11.3 to 12.5 | 4.6 | 4.3 to 5.0 | 0.6 | 0.5 to 0.7 |
|  |  |  |  |  |  |  |  |  |  |
| Age (in years) |  |  |  |  |  |  |  |  |  |
|  | 18 to 24 | 75.9 | 73.5 to 78.1 | 15.5 | 13.5 to 17.7 | 7.8 | 6.5 to 9.3 | 0.8 | 0.6 to 1.2 |
|  | 25 to 34 | 76.8 | 75.0 to 78.5 | 15.4 | 14.0 to 17.1 | 6.8 | 6.0 to 7.7 | 1.0 | 0.7 to 1.3 |
|  | 35 to 44 | 80.5 | 79.1 to 81.9 | 13.1 | 12.0 to 14.3 | 5.5 | 4.8 to 6.2 | 0.9 | 0.6 to 1.3 |
|  | 45 to 54 | 84.1 | 82.7 to 85.5 | 11.3 | 10.1 to 12.5 | 4.1 | 3.4 to 5.0 | 0.5 | 0.3 to 0.8 |
|  | 55 to 64 | 86.5 | 84.9 to 87.9 | 10.4 | 9.2 to 11.8 | 2.9 | 2.4 to 3.5 | 0.2 | 0.1 to 0.3 |
|  | ≥65 | 93.6 | 92.6 to 94.4 | 5.4 | 4.6 to 6.4 | 0.9 | 0.7 to 1.3 | 0.1 | 0.0 to 0.1 |
| Educational attainment |  |  |  |  |  |  |  |  |  |
|  | None or incomplete primary education | 90.8 | 90.0 to 91.6 | 5.7 | 5.1 to 6.4 | 2.9 | 2.5 to 3.3 | 0.6 | 0.4 to 0.9 |
|  | Complete primary or incomplete secondary education | 82.9 | 81.2 to 84.6 | 10.3 | 9.0 to 11.7 | 5.8 | 4.9 to 7.0 | 1.0 | 0.7 to 1.4 |
|  | Complete secondary or incomplete undergraduate course | 80.4 | 79.2 to 81.6 | 13.5 | 12.6 to 14.6 | 5.5 | 4.9 to 6.1 | 0.6 | 0.5 to 0.8 |
|  | University graduate | 72.6 | 70.6 to 74.4 | 21.6 | 19.8 to 23.4 | 5.5 | 4.8 to 6.5 | 0.3 | 0.2 to 0.5 |
| Marital status |  |  |  |  |  |  |  |  |  |
|  | Married | 87.3 | 86.3 to 88.1 | 9.7 | 9.0 to 10.6 | 2.7 | 2.3 to 3.1 | 0.3 | 0.2 to 0.4 |
|  | Not married | 80.0 | 79.2 to 80.9 | 13.2 | 12.5 to 14.0 | 6.0 | 5.5 to 6.4 | 0.8 | 0.6 to 0.9 |
| Skin Color |  |  |  |  |  |  |  |  |  |
|  | White | 80.9 | 79.7 to 82.0 | 14.5 | 13.5 to 15.5 | 4.2 | 3.8 to 4.8 | 0.4 | 0.3 to 0.6 |
|  | Black | 80.8 | 78.7 to 82.8 | 11.7 | 9.9 to 13.8 | 6.5 | 5.4 to 7.7 | 1.0 | 0.7 to 1.4 |
|  | Yellow | 86.6 | 79.7 to 91.3 | 9.8 | 5.6 to 16.7 | 2.7 | 1.4 to 5.1 | 0.9 | 0.2 to 4.0 |
|  | Brown | 85.4 | 84.5 to 86.2 | 9.4 | 8.7 to 10.1 | 4.6 | 4.2 to 5.1 | 0.6 | 0.5 to 0.8 |
|  | Indigenous | 89.3 | 81.3 to 94.2 | 7.5 | 3.2 to 16.2 | 2.3 | 1.2 to 4.5 | 0.9 | 0.2 to 3.9 |

Results include sample weights and control for survey design.

1 Defined as reporting having 0 g/day of pure alcohol in the past 30 days.

2 Defined as reporting having between 1 to 12.5 grams per day of pure alcohol in the past 30 days.

3 Defined as reporting having between 12.6 to 49.9 grams per day of pure alcohol in the past 30 days.

4 Defined as reporting having between ≥50 grams per day of pure alcohol in the past 30 days.

**Supplementary Table S3: STROBE Statement - Checklist of items that should be included in reports of *cross-sectional studies***

|  | Item N^o^ | Recommendation |
| --- | --- | --- |
| **Title and abstract** | 1 | (*a*) Indicate the study’s design with a commonly used term in the title or the abstract – Page 2 |
|  |  | (*b*) Provide in the abstract an informative and balanced summary of what was done and what was found - Page 2 |
| Introduction | | |
| Background/rationale | 2 | Explain the scientific background and rationale for the investigation being reported – Pages 3 and 4 |
| Objectives | 3 | State specific objectives, including any prespecified hypotheses – Page 5 |
| Methods | | |
| Study design | 4 | Present key elements of study design early in the paper – Page 5 |
| Setting | 5 | Describe the setting, locations, and relevant dates, including periods of recruitment, exposure, follow-up, and data collection – Page 5 |
| Participants | 6 | (*a*) Give the eligibility criteria, and the sources and methods of selection of participants – Page 5 |
| Variables | 7 | Clearly define all outcomes, exposures, predictors, potential confounders, and effect modifiers. Give diagnostic criteria, if applicable – Pages 6 and 7 |
| Data sources/ measurement | 8 | For each variable of interest, give sources of data and details of methods of assessment (measurement). Describe comparability of assessment methods if there is more than one group - Pages 6 and 7 |
| Bias | 9 | Describe any efforts to address potential sources of bias - Page 5 |
| Study size | 10 | Explain how the study size was arrived at – Page 5 |
| Quantitative variables | 11 | Explain how quantitative variables were handled in the analyses. If applicable, describe which groupings were chosen and why – Pages 6 and 7 |
| Statistical methods | 12 | (*a*) Describe all statistical methods, including those used to control for confounding – Page 7 |
|  |  | (*b*) Describe any methods used to examine subgroups and interactions - Page 7 |
|  |  | (*c*) Explain how missing data were addressed - NA |
|  |  | (*d*) If applicable, describe analytical methods taking account of sampling strategy - Page 7 |
|  |  | (*e*) Describe any sensitivity analyses - NA |
| Results | | |
| Participants | 13 | (a) Report numbers of individuals at each stage of study—eg numbers potentially eligible, examined for eligibility, confirmed eligible, included in the study, completing follow-up, and analysed – Page 20 |
|  |  | (b) Give reasons for non-participation at each stage - NA |
|  |  | (c) Consider use of a flow diagram - NA |
| Descriptive data | 14 | (a) Give characteristics of study participants (eg demographic, clinical, social) and information on exposures and potential confounders – Pages 7 and 8 |
|  |  | (b) Indicate number of participants with missing data for each variable of interest – Page 20 |
| Outcome data | 15 | Report numbers of outcome events or summary measures – Pages 7 and 8 |
| Main results | 16 | (*a*) Give unadjusted estimates and, if applicable, confounder-adjusted estimates and their precision (eg, 95% confidence interval). Make clear which confounders were adjusted for and why they were included – Pages 8, 9, 23 to 25 |
|  |  | (*b*) Report category boundaries when continuous variables were categorized – Pages 20 and 21 |
|  |  | (*c*) If relevant, consider translating estimates of relative risk into absolute risk for a meaningful time period - NA |
| Other analyses | 17 | Report other analyses done—eg analyses of subgroups and interactions, and sensitivity analyses – Pages 8 and 22 |
| Discussion | | |
| Key results | 18 | Summarise key results with reference to study objectives – Page 9 |
| Limitations | 19 | Discuss limitations of the study, taking into account sources of potential bias or imprecision. Discuss both direction and magnitude of any potential bias - Page 13 |
| Interpretation | 20 | Give a cautious overall interpretation of results considering objectives, limitations, multiplicity of analyses, results from similar studies, and other relevant evidence - Pages 9 to 14 |
| Generalisability | 21 | Discuss the generalisability (external validity) of the study results - Page 11 |
| Other information | | |
| Funding | 22 | Give the source of funding and the role of the funders for the present study and, if applicable, for the original study on which the present article is based - NA |
